# Fourth thoracic vertebra as landmark for depth of right internal jugular vein catheterization in infants

**DOI:** 10.1038/s41598-022-18787-4

**Published:** 2022-08-26

**Authors:** Guoliang Liu, Jianmin Zhang, Fang Wang, Heqi Liu

**Affiliations:** Department of Anesthesiology, Beijing Children’s Hospital, Capital Medical University, National Center for Children’s Health, No. 56 South Lishi Road, Xicheng District, Beijing, 100045 China

**Keywords:** Anatomy, Cardiology, Medical research

## Abstract

The carina is considered a reliable marker for the depth of right internal jugular vein catheterization in infants on chest radiograph. In adult anatomy, the carina is typically located at the level of the fifth thoracic vertebra. We are not aware of a positional relationship between infant carina and thoracic vertebrae. Thus, we evaluated that a vertebral body may be at the same level as carina and can be as radiographic landmarks for the depth of right internal jugular vein catheterization in infants. In this retrospective analysis, 108 infants (aged 1–12 months) who underwent congenital heart surgery between January 1, 2019 and June 30, 2019 were included. We analyzed the post-operative chest radiographs of those who underwent right internal jugular vein catheterization and assessed the positional relationship of the carina and vertebral bodies. We measured the vertical distance of the central venous catheter (CVC)  catheter tip from the carina (below the carina 22 mm, it may be close to or into the right atrium). In total, 95 children were enrolled; The carina was located at the third thoracic vertebra in two cases (2%) and at the fourth thoracic vertebra in 93 cases (98%). The distance between the tip of CVC and the carina was 10 (4, 15) mm, and 6.3% (6 cases) had the catheter tip at more than 22 mm below the carina. Most fourth thoracic vertebrae were at the same level as the carina on chest radiographs. Therefore, it has potential as a radiographic landmark for the depth of right internal jugular vein catheterization in infants on chest radiograph.

## Introduction

Central venous catheterization is an important cardiac surgery technique. The right internal jugular vein is the most used central venous catheter (CVC) placement site in infants; due to their relatively small size and short superior vena cava (SVC) length, determining catheterization depth requires a high level of precision to avoid arrhythmia, heart injury, and surgical interference^1,2^. When such catheterization is too shallow, it leads to inaccurate measurement of CVP and poor fluid and blood transfusion; the catheter may even be pulled out. An anatomical measurement of infant cadavers revealed that the carina is located 22 mm above the junction of the SVC and right atrium^3^, while clinical studies have revealed that the optimal depth of CVC tip placement is at the level of the carina on chest radiographs^3–8^. However, not all carina are particularly clear on chest radiographs, either for radiologic reasons or for patient reasons, and the position of carina varies with aspiration and affects the depth of puncture localization. In adult anatomy, the carina is typically located at the level of the fifth thoracic vertebra^9^. We are not aware of a positional relationship between infant carina and thoracic vertebrae. In this retrospective study, we aimed to analyze the relationship between the carina and thoracic vertebra in infants to determine the presence of a vertebral body, at the same level as the carina, that could be used as a radiographic landmark to guide the depth of right internal jugular vein catheterization in infants.

## Methods

This study was approved by the Ethics Committee of the Beijing Children’s Hospital, China (No. 2020-K-015). Informed patient consent was waived by the Ethics Committee of the Beijing Children’s Hospital. All the experiment protocol for involving human date and methods were in accordance with the guidelines of Declaration of Helsinki. Informed patient consent was waived due to the retrospective nature of the study.

Inclusion criteria: we retrospectively analyzed children aged between 1 and 12 months who underwent cardiac surgery and routinely took radiograph post-operation from January 1, 2019 to June 30, 2019. Exclusion criteria: ① vertebral deformity; ② no chest X-rays were taken after operation; ③ non-right internal jugular vein puncture and catheterization; ④ chest X-ray showed unclear central venous catheter.

**Figure 1:**
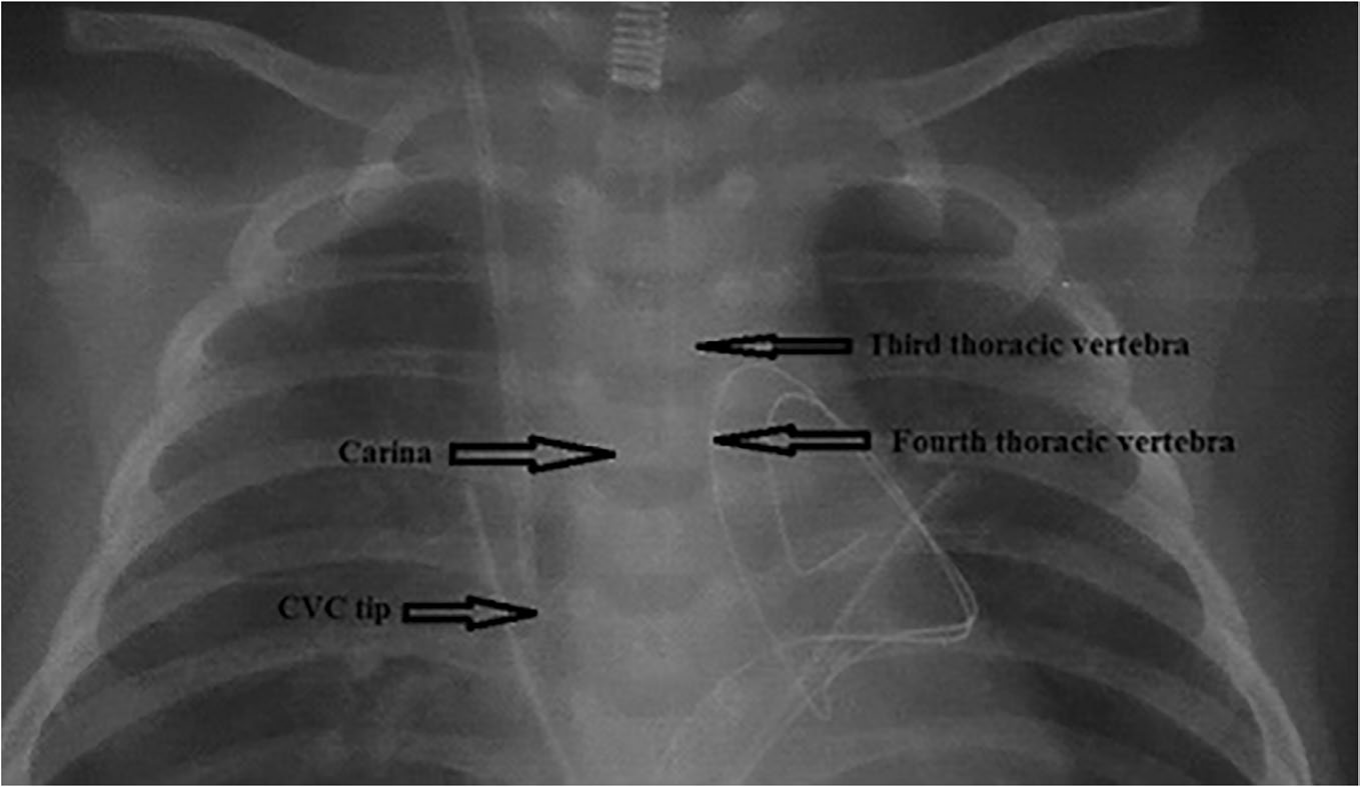
Carina, CVC tip and thoracic vertebra display on the radiograph. *CVC* central venous catheter.

The first post-operation chest radiograph of each child was analyzed using the Carestream Picture Archive and Communication System (PACS; Carestream Health Inc., Rochester, NY). We judge the positional relationship between the carina and the thoracic vertebrae on the chest X-ray mainly to watch which vertebral body it overlaps with carina. Finally, the vertebral body with the most overlap with the carina may be used as another marker for the depth of the internal jugular vein cannulation, as indicated in Fig. 1. We measured the vertical distance of the Central Venous Catheter (CVC) catheter tip from the carina (below the carina 22 mm, it may be close to or into the right atrium).

All measurements were performed by one experienced radiologist using the hospital's PACS software; all catheters were inserted under general anesthesia. Catheterization was performed by anesthesiologists, experienced in right internal jugular vein catheterization. Due to the retrospective nature of the study, details regarding catheter placement were not clear.

### Statistical analysis

Results are expressed as number of patients, percentage, mean ± standard deviation, and median (interquartile range-IQR) where appropriate. Percentage of carina located at thoracic vertebrae was determined by frequency descriptive statistics. Statistical analysis was performed with SPSS Statistics for Mac version 21 (IBM, Armonk, NY).

### Ethics approval and consent to participate

This study was approved by the Ethics Committee of the Beijing Children’s Hospital, China (No. 2020-K-015). Informed patient consent was waived by the Ethics Committee of the Beijing Children’s Hospital.

## Results

**Figure 2:**
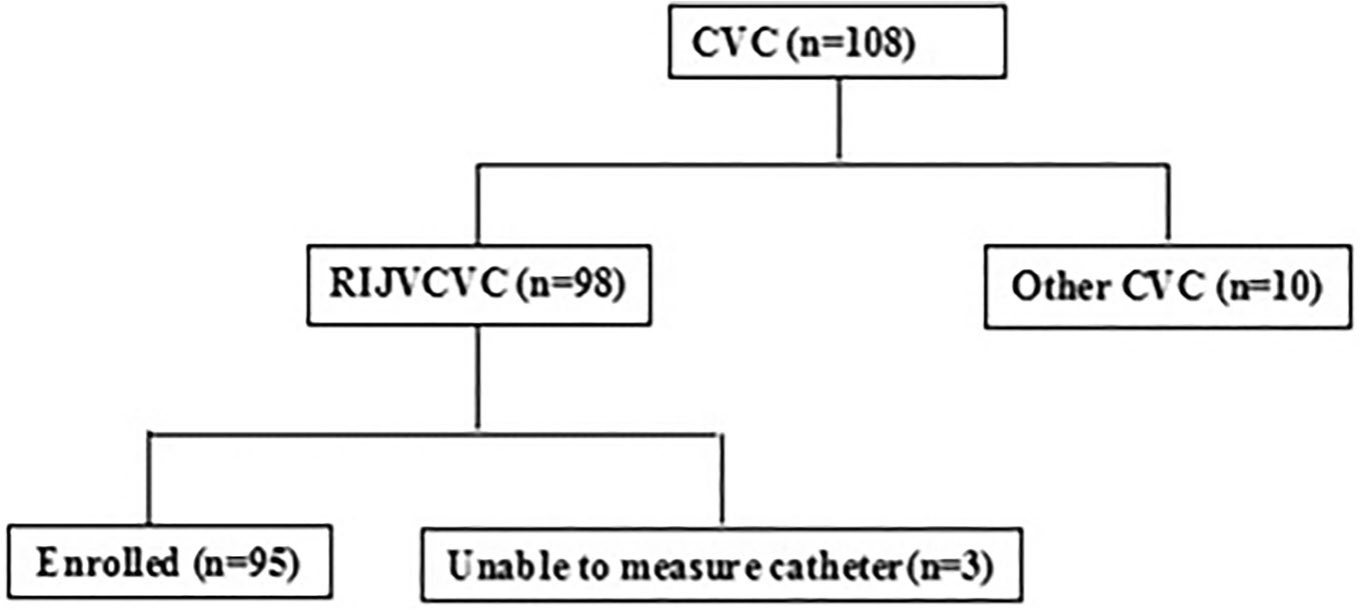
Details of assessed CVC cases. *CVC* central venous catheter, *RIJV* right internal jugular vein.

We reviewed the radiographs of 108 consecutive infants who underwent cardiac surgery; chest radiographs revealed that 98 of these patients underwent right internal jugular vein catheterization. Due to the overlap of the CVC and other cardiac leads, the data of three infants could not be assessed; therefore, we assessed 95 infants, as indicated in Fig. 2. The infants’ sex, age, weight, and medical diagnosis are summarized in Table 1.

**Table 1:** Infant demographic characteristics.

Variable	Value
Age, median (IQR), months	4 (3–5)
Weight, mean ± standard deviation, kg	5.6 ± 1.4
Sex, male/female, No. (%)	56/39 (59/41)
Diagnosis, No. (%)	66 (70)
**Ventricular septal defect**	
Atrial septal defect	2 (2)
Tetralogy of fallot	4 (4)
Total anomalous pulmonary venous connection	4 (4)
Patent ductus arteriosus	5 (5)
Aortic coarctation	5 (5)
Others	9 (10)

*IQR* interquartile range.

Analysis of the 95 cases the carina was located at the third thoracic vertebra in 2 case (2%), and the fourth thoracic vertebra in 93 cases (98%). The distance between the tip of CVC and the carina was 10 (4, 15) mm, and 6.3% (6 cases) had the catheter tip at more than 22 mm below the carina.

## Discussion

In this study, we retrospectively analyzed the postoperative chest radiographs of infants who underwent cardiac surgery and assessed the possibility of using a vertebral body as a radiographic landmark for the identification of the depth of right internal jugular vein catheterization. We found that the fourth thoracic vertebra is at the same level as the carina in most infants, and it can be a potential useful radiographic indicator of the depth of catheterization of the right internal jugular vein in infants on chest radiograph.

CVC insertion, including internal jugular vein catheterization, femoral vein catheterization, and subclavian catheterization, is a very important perioperative technique. Currently, the most widely utilized form is right internal jugular vein catheterisation^10^. The optimal timing of surgical treatment for children with certain congenital heart diseases is during infancy. Infants have a relatively large head and short neck; therefore, it is difficult to insert and place the right internal jugular vein catheter, especially in terms of the appropriate depth. There have been reports that improper CVC placement can cause serious complications in infants, such as pericardial effusions, cardiac tamponade, heart arrhythmias, artery punctures and hematomas^11–13^. In our study, we found the distance between the tip of CVC and the carina was 10 (4, 15) mm, and 6.3% (6 cases) had the catheter tip at more than 22 mm below the carina. Therefore, it can be seen that some catheters are placed too deep in our daily work, and there is the possibility of entering the right atrium. It is very meaningful for us to do this research.

According to current literature, the carina can be used as a reliable reference index for the depth of right internal jugular vein puncture on chest radiograph. However, we all know bone tissue is more recognizable than cartilage tissue under X-rays, so vertebral bodies are more visible clearly than the carina on chest radiographs. And the position of carina changes with the infants` breathing, but the position of the vertebral body does not. And in this study, we discovered that the fourth thoracic vertebra was at the same level as the carina in most cases (98%), contrary to what is observed in adults^9^; this discrepancy may be due to differences in the physical development stages of infants and adults. Our results suggest that the fourth thoracic vertebra can also be used as a radiographic marker for optimal CVC positioning, which may reduce complications.

There are certain limitations in this study. This is a retrospective study, some important information about the patient was not collected completely, such as height, the insertion length of the CVC and so on, resulting in some singleness of data in our analysis. Ninety-five children were a small sample size for a retrospective study, but a significant number for infants. Of course, we would like to consolidate our findings by including more infants in the future if possible. Finally, we only focused on infants; our results are therefore not necessarily applicable to children of all ages. These limitations highlight the need and scope for further, preferably prospective, studies on this topic.

## Conclusion

In conclusion, the fourth thoracic vertebra can be as a radiographic landmark for the depth of catheterization in the right internal jugular vein of infants on chest radiography. This feature may be valuable for reducing the clinical risks associated with inappropriate CVC placement.

## Data Availability

The datasets generated during and analysed during the current study are available from the corresponding author on reasonable request.
